# Book Review: Manual of Interventional Oncology: Manual of Interventional Oncology. Douglas M. Coldwell, Thieme, 2017, ISBN-13: 978-1626231382

**DOI:** 10.5334/jbsr.1568

**Published:** 2018-07-02

**Authors:** Francois Cousin

**Affiliations:** 1 CHU de Liège, BE

**Keywords:** Oncology, Intervention

## Abstract

The Manual of Interventional Oncology is a well-organized pocket book ideally suited for quick checking, designed to help the reader understand the main principles of cancer treatments and the role of interventional oncology.

The *Manual of Interventional Oncology* (cover, figure) is a well-organized pocket book ideally suited for quick checking, designed to help the reader understand the main principles of cancer treatments and the role of interventional oncology.

**Figure:**
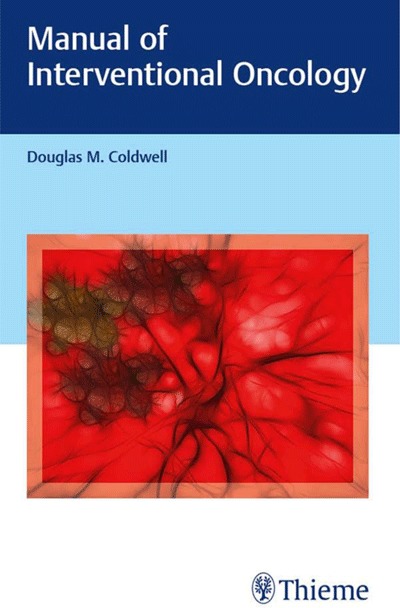
Book cover.

The first part of the book outlines the essentials of medical oncology, surgical oncology, and radiation oncology. In each of these three chapters, the fundamental principles of cancer treatments are shown and explained. This introduction provides a good overview of the multidisciplinary treatment of cancer and helps the interventional oncologist to take a more active role in this process. A quick overview of the essential procedures of interventional oncology is then given, from diagnosis to pain management, including therapeutic and palliative techniques.

The most-used chemotherapeutic agents are listed in a dedicated section, with practical details specified for each drug, such as mechanism of action, indications or side effects.

Pathology, epidemiology, genetics, staging, and treatment for the most frequent types of cancer are also covered in individual chapters, enhancing the potential role of interventional oncology for each tumour.

To conclude the book, the author uses his great experience in the field of interventional oncology to provide nine enlightened pieces of advice as a road map to build and develop your interventional oncology practice and to facilitate your integration into the tumour therapy process within the clinical team.

In order to be actively involved in medical oncology groups (which is essential in the field of oncology), it is of paramount importance to understand cancer comprehensively, from genetics to treatments. Unfortunately, this knowledge is not usually fully provided during radiology residency, but this book provides a concise description of the clinical information you need to fill that gap, and it is a solid foundation on which to build your interventional oncology practice. The reader will not find exhaustive descriptions or advanced technical details concerning interventional oncology procedures in this book, but he will learn about numerous non-radiological fundamentals concerning oncology that will be beneficial in improving an interventional oncologist’s skills.

In summary, the *Manual of Interventional Oncology* is a very useful and practical pocket tool, recommended not only to interventional radiologists but to all radiologists who are interested in cancer imaging.

